# Immobilization of Laccase in Alginate-Gelatin Mixed Gel and Decolorization of Synthetic Dyes

**DOI:** 10.1155/2012/823830

**Published:** 2012-07-31

**Authors:** Mehdi Mogharabi, Nasser Nassiri-Koopaei, Maryam Bozorgi-Koushalshahi, Nastaran Nafissi-Varcheh, Ghodsieh Bagherzadeh, Mohammad Ali Faramarzi

**Affiliations:** ^1^Department of Pharmaceutical Biotechnology, Faculty of Pharmacy and Biotechnology Research Center, Tehran University of Medical Sciences, P.O. Box 14155–6451, Tehran 14174, Iran; ^2^Department of Chemistry, Faculty of Sciences, University of Birjand, Birjand 9717853577, Iran; ^3^Department of Pharmaceutical Biotechnology, School of Pharmacy, Shahid Beheshti University of Medical Sciences, Tehran 1457795343, Iran

## Abstract

Alginate-gelatin mixed gel was applied to immobilized laccase for decolorization of some synthetic dyes including crystal violet. The immobilization procedure was accomplished by adding alginate to a gelatin solution containing the enzyme and the subsequent dropwise addition of the mixture into a stirred CaCl_2_ solution. The obtained data showed that both immobilized and free enzymes acted optimally at 50°C for removal of crystal violet, but the entrapped enzyme showed higher thermal stability compared to the free enzyme. The immobilized enzyme represented optimum decolorization at pH 8. Reusability of the entrapped laccase was also studied and the results showed that *ca*. 85% activity was retained after five successive cycles. The best removal condition was applied for decolorization of seven other synthetic dyes. Results showed that the maximum and minimum dye removal was related to amido black 10B and eosin, respectively.

##  Introduction

While traditional methods in chemical processes have improved in the last decades, extensive attention has been paid to alternative techniques that utilize enzymes involving excellent characteristics, such as high activity, selectivity, and specificity. In addition, enzymes action at mild conditions of pH, pressure, and temperature proposes them as candidates for suitable catalysts in industries where low cost, energy savings, and simplicity are important [1, 2]. However, despite these advantages, some practical problems restrict their use, such as the high-cost isolation and purification process and instability in organic media and high temperatures. To overcome these limitations, several methods have been suggested and the most important of which are immobilization techniques [3, 4]. Enzyme entrapment uses natural and synthetic polymers, such as agarose, agar, and gelatin, through thermoreversal polymerization alginate, polyvinyl acetate, acrylic acid, and *β*-carrageenan by ionotropic gelation [5].

Gelatin consists of proteins and peptides produced by the denaturation of collagen, which breaks down into smaller fragments. Due to its unique physical properties, such as a melting point close to physiological temperature, gelatin is used in a variety of applications, especially in the food and pharmaceutical industries [6]. Gelatin immobilization methods have been developed for entrapment of microbial cells and enzymes, especially when the enzyme is placed in a whole cell. While the gelation process is reversible with temperature and displays no efficient immobilization, other than at 30–35°C, some methods have been investigated to achieve an irreversible gelation process, such as using cross-link agents [7, 8]. Single-step immobilization is one of the most frequently used methods of entrapment, which could be performed by simple gelation through lowering or raising temperatures of biopolymers such as agar, agarose, *κ*-carrageenan, and chitosan. Although it is easy to achieve, but this kind of preparation suffers from low mechanical strength and heat damages. Therefore, alternative methods are required to produce a more porous structure with higher mechanical stability and desirable elastic behavior. There are also some techniques for stabilization of alginate suggested by Birnbaum and colleagues which consists the treatment of alginate beads with polyethyleneimine-HCl, activation of alginate with a mixture of 1-ethyl-3-(3-dimethyl-amino-propyl)-carbodiimide and N-hydroxysuccinimide, and addition of sodium metaperiodate to alginate [9].

Laccases (benzenediol : oxygen oxidoreductases, E.C. 1.10.3.2) are an interesting group of multicopper enzymes produced by higher plants and fungi that catalyze the oxidation of a wide range of organic compounds, such as phenols, in the presence of molecular oxygen [10, 11]. The most important applications discussed for laccases include pharmaceutical and food industries, textile effluent transformation, and wastewater detoxification biosensors. The unique properties of laccases, such as high stability in solution, mild reaction conditions, and selectivity for phenolic structure, make them attractive for use in chemical synthesis [12]. The mechanism of phenolic ring oxidation by laccase has been previously described. Releasing molecular nitrogen, instead of the formation of low molecular weight aromatic amines that are easily absorbed through the skin and known as powerful carcinogens and mutagens, could be considered as an advantage for using laccase for detoxification of synthetic dyes especially aromatic azo dyes [13].

Wastewater released by various industries that use synthetic dyes because of their low cost, ease of synthesis, and color variety can pollute and harm the aquatic environment. Decolorization of synthetic dyes by using special biocatalysts has received great attention because of efficient decolorization and nontoxic product generation [14, 15]. Crystal violet is a widely used synthetic pigment for dye processing. It is stable and nondegradable by conventional methods and has been identified as a main factor of industrial effluent contamination. Textile factories are under pressure to apply environmental-friendly technologies to remove it [16].

The aim of the present study was to apply alginate-gelatin mixed gel to immobilize laccase, which is further employed in the decolorization of some synthetic dyes, such as crystal violet, in aqueous solutions. Optimum pH, temperature, and proper enzyme content for decolorization by the immobilized laccase were also studied. To our knowledge, the use of alginate-gelatin mixed gel has not been examined previously for the purpose of enzyme entrapment.

##  Experimental

### 2.1. Chemicals and Instruments

 Crystal violet, coomassie blue G-250, bromothymol blue, amido black 10B, methyl red, eosin, and malachite green (Table 1) were purchased from Sigma-Aldrich (St. Louis, MO, USA). Gelatin and sodium alginate were obtained from Merck (Darmstadt, Germany). All other reagents and chemicals were of the highest purity available. Extracellular laccase was purified from the submerged fermentation of the soil isolate ascomycete *Paraconiothyrium variabile* [10, 17, 18]. Decolorization was monitored by UV-VIS spectroscopy. Absorbance was scanned by a double-beam UV-Vis 2501 PC spectrophotometer (Shimadzu, Japan). The mechanical force to break the beads was measured with a tensiometer (Zwick/Roell 030, Germany). Optical images of the gelatin-alginate beads were taken with a USB Digital Microscope Pro (Dino-Lite, Taiwan). Scanning electron microscope (SEM, Hitachi S-2400, Japan) was applied for studying the size and surface of the beads.

### 2.2. Preparation of Mixed Gels, Immobilization of Laccase, and Decolorization of Crystal Violet

The gelatin-alginate mixed gel was prepared according to the method of Panouille and Larreta-Garde [19], with some modifications. Briefly, entrapment of laccase in gelatin-alginate mixed gel was performed by adding 0.1 g sodium alginate to 10 mL of a solution containing 10% (w/v) gelatin and laccase in the range of 5–50 mg (1 mg enzyme is equal to 1.25 U) under continuous stirring at room temperature. The mixture was syringed into a stirring CaCl_2_ solution (200 mM), and the resulting beads were left to be harden for 1 h under the same conditions, washed three times by deionized water, and then stored at 4°C prior to being used in the decolorization study. The amount of bound protein was determined by Bradford's method [20], using the following equation: *Q* = (*Ci* − *Cf*)/*mV*, where *Q* was the bound enzyme (mg enzyme/g beads), *Ci* and *Cf* were the initial and final enzyme concentrations in the solution (mg/mL), *V* was the volume of the solution (mL), and *m* was the mass of the beads (g). The immobilized laccase (10 g) was transferred into a 20 mL buffer solution, at pH 3–9, containing crystal violet (final concentration 0.083 mM), at a 30–70°C temperature range, for 20 min. Maximum absorbance was monitored according to *λ*_max⁡_ of the dye (Table 1). Relative decolorization was calculated by the following equation: relative decolorization (%) = (*A*_initial_ − *A*_final_)/*A*_initial_× 100, where *A*_initial_ was the initial absorbance and *A*_observed_ was the final absorbance at the given wavelength.

### 2.3. Optimum Temperature, PH, and Enzyme Concentration of the Immobilized Laccase

 The effect of pH on the enzymatic decolorization was monitored with a dye concentration of 0.25 mg/mL at a 3–9 pH range adjusted by citrate or ammonia buffers. To determine the effect of temperature on the enzymatic decolorization, the reaction mixture was incubated at a thermal range of 30–70°C, in steps of 10°C. In order to assess the effect of enzyme quantity on decolorization, the reaction was started with different enzyme amounts, from 0.25 to 0.5 mg/mL, in 0.05 mg/mL increments. The experiments were performed in triplicate; the results shown are means ± standard deviation.

### 2.4. Mechanical Strength, Optical Image, and SEM

The force required to rupture the beads was recorded by compressing the beads using a tensiometer; the results are the average force from 20 independent tests. The optical microscopy was used to evaluate the shape, surface, and size of the beads, and scanning electron microscopy (SEM) was also applied to study the structure of the fabricated beads.

### 2.5. Reusability

Reusability of the gelatin-alginate immobilized enzyme was investigated in acetate buffer solution 0.1 M at pH 8 and 45°C. The used beads were filtered at the end of each cycle and washed three times with the same buffer to treat in the next fresh colored solution. The reusability study was performed in triplicate.

### 2.6. Decolorization of Synthetic Dyes

Decolorization of the other dyes from the aqueous solution—coomassie blue G-250 (0.086 mM), bromothymol blue (0.095 mM), amido black 10B (0.045 mM), methyl red (0.125 mM), eosin (0.038 mM), and malachite green (0.112 mM)—was investigated by adding 10 g immobilized laccase to 20 mL citrate buffer solution (0.1 M, pH 4.5) containing dye, at 45°C for 20 min. Relative decolorization was calculated as described above for crystal violet. Some properties of the applied synthetic dyes and the percentages of dye removal are shown in Table 1. Standard deviation and mean of the results from three independent experiments were calculated using SigmaPlot for Windows (version 10.0).

##  Results and Discussion

### 3.1. Effect of Experimental Parameters on Crystal Violet Decolorization

**Figure 1:**
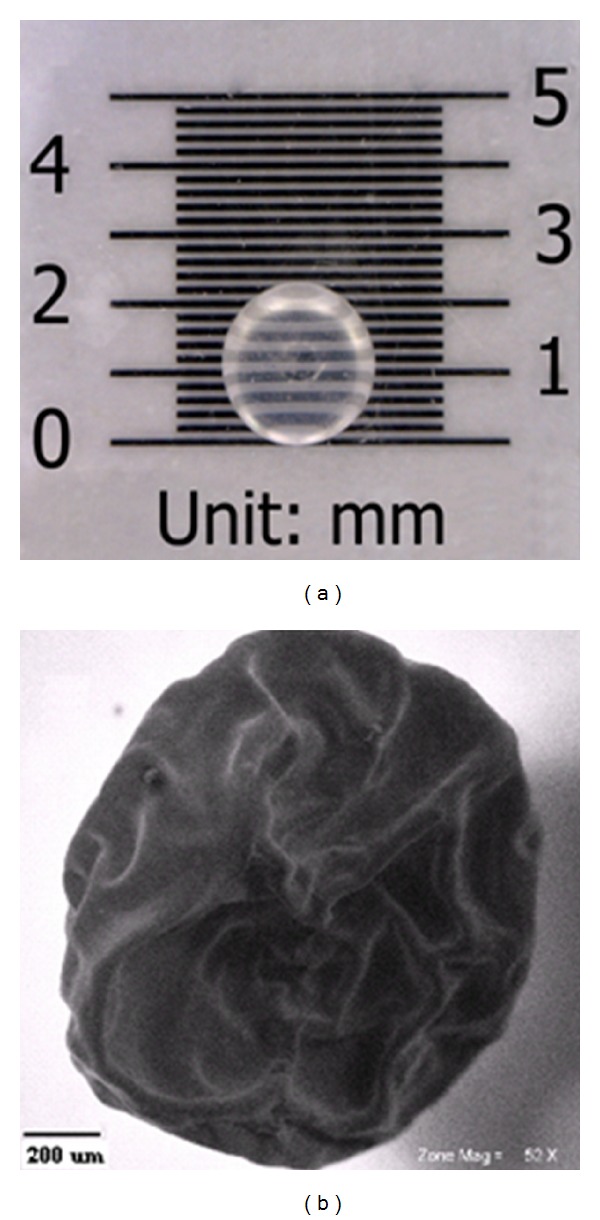
Optical image (a) and SEM of the prepared gelatin-alginate bead (b).

 Spherical and regular-shaped gelatin-alginate beads were obtained by using ionotropic gelation; the diameters of the beads were 2.0–2.5 mm (Figure 1(a)). The results, as shown in Table 2, indicated the rupture force increase with the rise in alginate concentration. The prepared beads containing 5% alginate exhibited the highest mechanical stability. Alginate could provide a very strong network that required 0.204 kgf force to rupture. Earlier studies had also reported a direct correlation between rupture force and alginate concentration [21].

**Figure 2:**
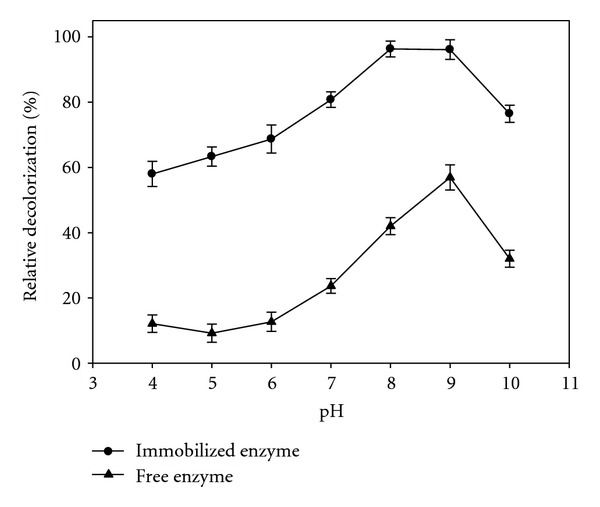
Effect of pH on relative decolorization activity of free laccase (▲) and immobilized laccase (●). Data were mean values ± SD.

Among the investigated parameters, pH plays the key role in dye decolorization. The effect of pH on dye decolorization was examined at pH ranging from 3 to 9, using acetate (pH 3–6) and ammonia (pH 7–9) buffers. The crude enzyme significantly displayed higher decolorization activity in basic pH (Figure 2) with a sharp increase at pH values above 6 and maximum activity at pH 9. The optimal pH for the immobilized enzyme activity was 8. It shows that pH of the environment has significant effects on decolorization which suggests that the mass transfer in the gel matrix may depend on the transport of bulk H^+^ ion into the gel matrix [22].

**Figure 3:**
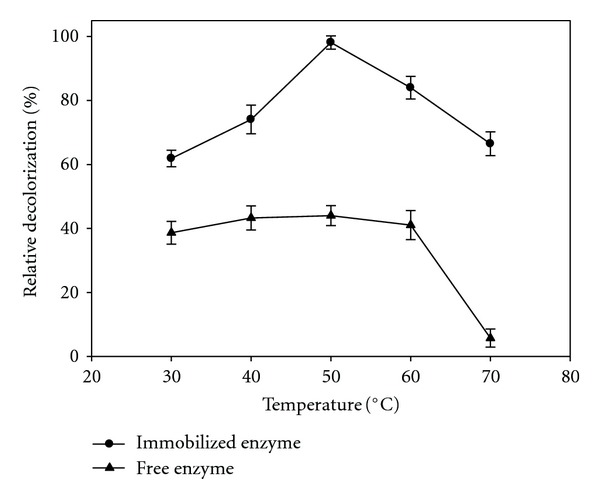
Influence of temperature on relative decolorization activity of free laccase (▲) and immobilized laccase (●). Data were mean values± SD.

The effect of temperature on dye decolorization was investigated by changing the reaction mixture temperatures in a 30–70°C range. The results obviously showed that decolorization increased as the temperature raised up to 50°C; at higher temperatures, decolorization efficiency remarkably decreased, which normally attributed to denaturation of enzyme. However, the comparison of decolorization results indicated high thermal stability of immobilized enzyme and confirmed the protective role of alginate-gelatin mixed gel for maintaining enzyme activity. At 50°C, the crude and immobilized enzymes exhibited more activity, as shown in Figure 3. Similarly, Forootanfar and colleagues [10] reported that the optimum temperature for laccase activity was 50°C. Other research studies have reported an optimum temperature range of 45–50°C for laccase activity immobilized by both covalent binding and adsorption [23, 24].

**Figure 4:**
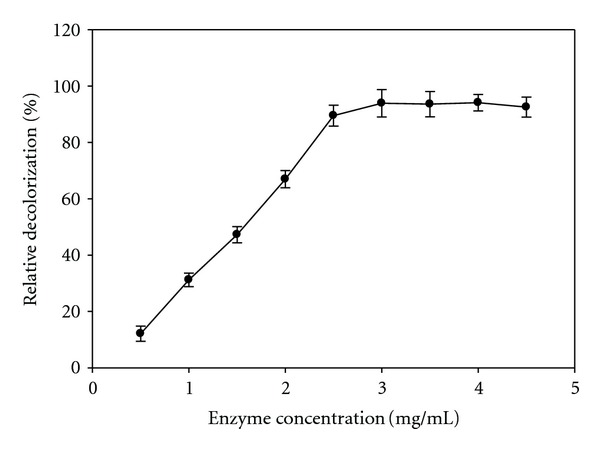
Effect of enzyme concentration on relative decolorization activity of the immobilized laccase. Data were mean values ± SD.

To determine the proper amount of enzymes required for maximum decolorization, the effect of enzyme quantity on dye decolorization was also studied. As shown in Figure 4, decolorization increased as enzyme quantity increased, from 0.5 to 5 mg/mL. However, the results demonstrated that the minimum enzyme quantity to obtain maximum decolorization was 2.5 mg/mL.

The optimum conditions were obtained as described above and applied for the decolorization of other synthetic dyes (Table 1). It is notable that the efficiency of laccase enzyme for removal and detoxification of these synthetic dyes has been previously reported and discussed [13, 25, 26]. The results showed that amido black 10B and eosin exhibited maximum and minimum dye removal, respectively. It was observed that the immobilized laccase was able to decolorize more than 80% of amido black 10B, while Selvam and colleagues [27] reported 15% decolorization by laccase. However, all of the dyes were oxidized at the maximum rate in pH 8. Kinetic studies (Table 1) suggested that among the used dye substrates for the enzymatic removal, methyl red and malachite green are preferred, and, in contrast, eosin is a poor substrate [13].

### 3.2. Reusability of Immobilized Laccase

**Figure 5:**
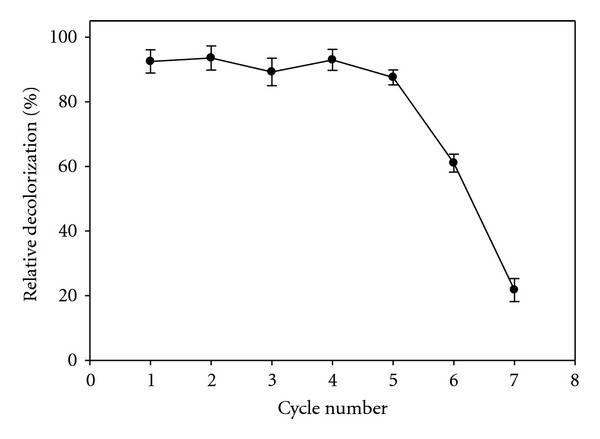
Reusability of immobilized laccase in the reaction condition. Data were mean values± SD.

 Reusability of immobilized enzymes exhibits the most important aspect for industrial applications, because immobilization of enzymes decreases the cost of production due to their repeated continuous uses. In this work, reusability of the immobilized enzyme was investigated up to seven cycles; the residual activities are presented in Figure 5. During enzymatic reactions, the alginate-gelatin mixture may cause a decrease in the pore sizes of the network, leading to difficulties in the diffusion of the substrate and product in the matrix of the gel. This restriction may cause a decrease in the efficient activity of laccase entrapped in gel after repeated use. In the literature, there are reports of successful reuses of various immobilized laccase systems, such as 60% activity after ten cycles for covalently immobilized laccase on activated polyvinyl alcohol [28–30] and 80% after five cycles for laccase immobilized on amine-terminated magnetic nanocomposites by cross-linking method [31].

##  Conclusions

Immobilization of laccase has received increasing attention in dye removal of aqueous solutions such as wastewater. Gelatin-alginate beads were prepared as a matrix system for laccase entrapment; the immobilized enzymes exhibited more stability during operation compared to free enzymes, a result that can be considered an advantage in wastewater treatment. In addition, the reusability of gelatin-alginate beads provides economic benefits when used in large-scale applications. Future investigations may focus on the decolorization of different types of dyes that are widely used in the chemical and textile industries.

## Figures and Tables

**Table 1:** Names, classification, maximum absorbance, and removal percentage of eight synthetic dyes using immobilized enzymes (in citrate buffer 0.1 M, pH 4.5).

Name	Classification	*λ*_max⁡_	Dye removal (%)	Removal rate (nmol/min)^b^
Amido black 10B	Diazo	618	86.9 ± 1.3^a^	22.5 ± 2.3
Bromothymol blue	Triphenylmethan	430	53.8 ± 1.2	27.2 ± 1.4
Coomassie blue G-250	Triphenylmethan	575	71.4 ± 0.8	53.8 ± 1.5
Crystal violet	Triphenylmethan	595	58.1 ± 1.5	95.3 ± 2.8
Eosin	Heterocyclic	517	32.8 ± 1.2	20.1 ± 1.2
Malachite green	Triphenylmethan	620	76.3 ± 2.1	120.7 ± 2.5
Methyl green	Triphenylmethan	630	77.5 ± 1.5	73.4 ± 1.4
Methyl red	Azo	522	52.1 ± 1.8	147.3 ± 2.8

^a^Mean ± SD (*n* = 3); ^b^Kinetic study was performed by monitoring the loss of absorbance at *λ*_max⁡_.

**Table 2:** Maximum force required to rupture beads and the amount of bound protein.

Alginate	Maximum force (kgf)	Bound protein (mg/g carrier)
1%	0.125 ± 0.033^a^	0.063 ± 0.035^b,c^
2%	0.152 ± 0.041	0.094 ± 0.043
3%	0.161 ± 0.037	0.127 ± 0.080
4%	0.177 ± 0.047	0.153 ± 0.075
5%	0.204 ± 0.063	0.166 ± 0.098

^a^Mean ± SD (*n* = 20); ^b^Mean ± SD (*n* = 3); ^c^Beads was washed with 50 mM citrate buffer (pH 5.0).
